# Comparison of diffuse correlation spectroscopy, interferometric diffusing wave spectroscopy, and speckle contrast optical spectroscopy for blood flow monitoring

**DOI:** 10.1117/1.NPh.12.3.035005

**Published:** 2025-08-12

**Authors:** Joseph B. Majeski, Rodrigo M. Forti, Sang Hoon Chong, Santosh Aparanji, Mingjun Zhao, Kenneth Abramson, Nithin R. Ramachandran, Vivek J. Srinivasan, Wesley B. Baker, Arjun G. Yodh

**Affiliations:** a University of Pennsylvania, Department of Physics and Astronomy, Philadelphia, Pennsylvania, United States; b Children’s Hospital of Philadelphia, Division of Neurology, Philadelphia, Pennsylvania, United States; c Boston Children’s Hospital, Harvard Medical School, Department of Radiology, Boston, Massachusetts, United States; d New York University, Tech4Health Institute, Langone Health, New York City, New York, United States

**Keywords:** diffuse optics, blood flow monitoring, diffuse correlation spectroscopy, speckle contrast optical spectroscopy, interferometric diffusing wave spectroscopy

## Abstract

**Significance:**

Noninvasive optical measurements of blood flow have many applications. Measurements have been demonstrated with diffuse correlation spectroscopy (DCS), interferometric diffusing wave spectroscopy (iDWS), and speckle contrast optical spectroscopy (SCOS) techniques, but concurrent measurements with all three techniques in the same experiment have not been compared.

**Aim:**

We aim to evaluate the comparative strengths and weaknesses of SCOS, iDWS, and DCS methods in controlled experiments.

**Approach:**

We performed *in vitro* temperature-controlled microsphere flow phantom and *in vivo* arm cuff occlusion experiments using SCOS, iDWS, and DCS concurrently and in the same geometry.

**Results:**

*In vitro* results showed absolute flow metrics agreement between iDWS and DCS and demonstrated large gains in signal-to-noise for iDWS and SCOS compared with DCS; relative changes in flow measured by SCOS were also in good agreement with DCS and iDWS. The *in vivo* cuff occlusion results showed agreement of relative changes in flow measured by DCS, iDWS, and SCOS. However, DCS recovered a flow pulsatility index that was larger than iDWS and SCOS indices.

**Conclusions:**

The experiments demonstrate the equivalency of absolute flow measures from iDWS and DCS and improved precision of pulsatile waveforms from SCOS. These results emphasize the need for rapid development and adoption of iDWS and SCOS.

## 1 Introduction

The speckle fluctuations of diffuse light facilitate noninvasive measurement of blood flow in deep tissues at the bedside. Until recently, the predominant technique employed for this purpose has been diffuse correlation spectroscopy (DCS). The traditional DCS methodology obtains the temporal intensity autocorrelation function of one or a few speckles of diffuse light and models transport of the electric field temporal correlation function through tissue with the correlation diffusion equation.^1^^,^^2^ DCS can then extract tissue blood flow information by fitting the autocorrelation function decay rate of light intensity to solutions of the correlation diffusion equation.^2^^–^^9^

Since its introduction, DCS has been used to directly monitor (and image) cerebral blood flow in many human studies. For example, DCS has assessed cerebral blood flow in adults during functional activation,^4^^,^^10^^,^^11^ in patients after brain injury,^12^^–^^19^ and in studies of cognitive impairment.^20^ It has also been used to monitor cerebral blood flow in the first weeks of life,^21^^,^^22^ in neonates with congenital heart disease,^23^^–^^26^ and in children with hydrocephalus.^27^^,^^28^ In addition to brain, investigations have employed DCS to monitor cancer therapy,^29^^–^^33^ skeletal muscle physiology,^6^^,^^34^^–^^36^ spinal cord injury,^37^ and bone health.^38^^,^^39^ DCS signals have been collected at the bedside over long timescales, i.e., hours to days,^16^^,^^18^^,^^40^ as well as rapid timescales to resolve pulsatile flow variations.^41^^–^^44^ Importantly, DCS measurements of blood flow have been validated against measurements by standard medical diagnostics such as PET,^45^ MRI,^6^^,^^46^ Xe-CT,^12^ and ultrasound.^22^^,^^47^ Despite these successes, the number of collected speckles is typically on the order of 4 for traditional DCS measurements. This limitation impacts achievable signal-to-noise ratio (SNR), and consequently, depth penetration is limited because the SNR sets the maximum source-detector separation to be ∼2.5  cm.

Recently, highly parallel variants of the basic photon correlation technique have been demonstrated.^48^^–^^51^ Herein, we focus on two techniques that hold promise to dramatically improve SNR and hence the depth penetration of diffuse correlation methods: speckle contrast optical spectroscopy (SCOS) and interferometric diffusing wave spectroscopy (iDWS). SCOS and iDWS can provide orders of magnitude improvement in SNR due to their highly parallelized detection of hundreds or even millions of speckles.^52^^–^^56^ SCOS utilizes large-area complementary metal-oxide-semiconductor (CMOS) or charge-coupled device (CCD) sensors to capture short exposure images that can contain millions of speckles, the spatial/temporal intensity variations of which, quantified by speckle contrast, can be related to the DCS blood flow index.^57^^,^^58^ iDWS uses a Mach–Zehnder interferometer geometry for coherent amplification of the speckle field, and it uses a fast line scan camera for parallelized detection of hundreds of speckles. iDWS directly recovers the temporal electric field autocorrelation function, which permits determination of the DCS blood flow index.^53^

Although these new methods show great promise compared with the traditional DCS approach, to our knowledge, a full comparison study comparing all three methods in the same experiment has not yet been reported in the literature. Such stake-in-the-ground comparisons can help inform the assessment of data equivalency, quantification of SNR improvement, and elucidation of limitations. This information, in turn, will enable researchers to make decisions about which methodology is best suited for their specific application. To this end, herein we report on *in vivo* and *in vitro* measurements simultaneously performed with SCOS, DCS, and iDWS.

## 2 Methods

### 2.1 Correlation Diffusion Equation

DCS, iDWS, and SCOS provide information on tissue blood flow by quantifying fast intensity fluctuations of coherent light that arise from the interaction of photons with dynamic scatterers such as red blood cells.^3^^,^^53^^,^^58^ When light diffuses through tissue, it experiences absorption events due to interaction with endogenous chromophores, and it experiences scattering events from endogenous static scatterers and moving scatterers. This phenomenon is modeled by the correlation diffusion equation (CDE)^3^^,^^7^^,^^8^

$$[\nabla \cdot (D(r)\nabla )-v\mu_{a}(r)-\frac{\alpha }{3}v\mu_{s}^{\prime }(r)k_{0}^{2}\langle {\Delta r}^{2}(\tau )\rangle ]G_{1}(r,\tau )=-vS(r).$$ (1)

Here, $G_{1}(r,\tau )=|\langle E(r,t)\cdot E^{*}(r,t+\tau )\rangle |$ is the absolute value of the temporal autocorrelation function at position r and with delay-time τ of the unnormalized electric field (E(r,t)=e^E(r,t)ei(ωt+ϕ(r,t))), where e^ represents the unit vector in the direction of polarization, and unbolded E is the amplitude of the field); S(r) is a source term, $D(r)=v/[3(\mu_{a}(r)+\mu_{s}^{\prime }(r))]$ is the photon diffusion coefficient, $\mu_{a}(r)$ is the tissue absorption coefficient, $\mu_{s}^{\prime }(r)$ is the tissue reduced scattering coefficient, v is the speed of light in the medium; $k_{0}=2\pi n_{o}/\lambda$ is the wave number in the medium that depends on the tissue index of refraction ($n_{o}$) and the wavelength of light (λ); α is the fraction of scatterers in the medium that are moving, and $\left\langle {\Delta r}^{2}(\tau )\right\rangle$ is the mean square displacement of the moving scatterers in delay time τ.

For *in vivo* human and animal applications, the largest contributor to dynamic fluctuations of light intensity are red blood cells.^59^ Red blood cells in the vasculature experience Brownian-like motion, i.e., with $\langle \Delta r(\tau {)}^{2}\rangle =6D_{b}\tau$, where $D_{b}$ is an effective Brownian diffusion coefficient that can be empirically related to an average red blood cell displacement in the vasculature.^60^^–^^62^ Importantly, multiple studies have demonstrated the proportionality of the blood flow index, $BF_{i}$ to true blood flow;^6^^,^^12^^,^^22^^,^^45^^–^^47^^,^^60^ here, $BF_{i}=\alpha D_{b}$, where α is the fraction of photon scattering events that occur from moving red blood cells as noted above. In monitoring applications, biological tissue is often modeled as a semi-infinite homogeneous medium, with point sources and point detectors in the same plane on the tissue surface; the point source and detector are separated by a distance ρ. In this case, the solution to the CDE^7^ is

$$G_{1}(\tau )=\frac{v}{4\pi D}[\frac{e^{-K(\tau )r_{1}}}{r_{1}}-\frac{e^{-K(\tau )r_{2}}}{r_{2}}].$$ (2)

Here, $K(\tau )=\sqrt{3\mu_{a}(\mu_{a}+\mu_{s}^{\prime })+(\mu_{a}+\mu_{s}^{\prime })\mu_{s}^{\prime }k_{0}^{2}6\alpha D_{b}\tau }$, $r_{1}=\sqrt{\rho^{2}+z_{o}^{2}}$ and $r_{2}=\sqrt{\rho^{2}+(z_{o}+2z_{b}{)}^{2}}$, $z_{o}=1/\mu_{s}^{\prime }$, and $z_{b}=[2(1+R_{\mathrm{eff}})]/[3\mu_{s}^{\prime }(1-R_{\mathrm{eff}})]$. $R_{\mathrm{eff}}$ is the effective Fresnel reflection coefficient dependent upon the refractive index of tissue ($n_{o}$) and the external medium (n).

In traditional DCS, $BF_{i}$ is derived from the temporal intensity fluctuations of a single (a few) speckle(s) collected by a single- or few-mode fiber. This fiber is coupled with a single-photon detector such as a single-photon avalanche diode (SPAD) or photomultiplier tube. Traditional DCS does not measure the electric field temporal autocorrelation function directly. Rather, DCS measures the normalized intensity temporal autocorrelation function, $g_{2}(\tau )$, defined as $g_{2}(\tau )=\langle I(t)I(t+\tau )\rangle /{\left\langle I(t)\right\rangle }^{2}$. This intensity autocorrelation function can be related to the normalized electric field autocorrelation function, $g_{1}(\tau )=G_{1}(\tau )/G_{1}(0)$, through the Siegert relation, $g_{2}(\tau )=1+\beta_{\mathrm{DCS}}|g_{1}(\tau ){|}^{2}$. $\beta_{\mathrm{DCS}}$ is a coherence parameter that depends on the source and detector properties. Very briefly, to extract tissue $BF_{i}$ with traditional DCS, one typically performs a nonlinear regression fit of the measured $g_{2}(\tau )$ to the semi-infinite homogeneous solution of the CDE given in Eq. (2) (and using Siegert’s relation).

Like DCS, iDWS collects fast intensity fluctuations arising from dynamic scattering events. The measured signal ($I_{\text{total}}(t)$) is a result of the coherent mixing of signals from the sample and reference arm. Note that for simplicity the derivation below assumes the light in the sample and reference arm are linearly co-collimated and are co-polarized, traveling in the e^ direction. Therefore, we drop the vector notation. We have also dropped the dependence on detector position (r) from the expressions for intensity and field. This coherent mixing gives^53^^,^^63^

$$I_{\text{total}}(t)=|E_{S}(t)e^{i(\omega t+\phi_{S}(t))}{|}^{2}+|E_{R}(t)e^{i(\omega t+\phi_{R}(t))}{|}^{2}\;+2\text{Re}\{E_{S}(t)e^{i(\omega t+\phi_{s}(t))}E_{R}(t)e^{i(\omega t+\phi_{R}(t))*}\},$$ (3)

where $E_{S}$ and $E_{R}$ are the amplitudes of the sample and reference fields, respectively; $\phi_{S}$ and $\phi_{R}$ are their respective phases; ω is the angular frequency of the source laser used, and t is time. The use of this interferometric detection scheme enables recovery of the temporal electric field autocorrelation function of the weak sample light via coherent amplification with the reference beam.^54^ We assume the sample light signal is small compared with the reference light signal, the variation in the total electric field is predominately due to phase fluctuations, and the fixed pathlength of the reference arm results in negligible phase changes for the reference field. We then approximately rewrite the total intensity as

Itotal(t)≈ER2+2Re{ES(t)ei(ωt+ϕs(t))ERei(ωt+ϕR)*}. (4)

From this total intensity, a rolling average is taken over a period much greater than the sample’s characteristic decorrelation time, and this rolling average is subtracted from $I_{\text{total}}(t)$. As the interference term is an alternating current waveform and the reference arm is constant, this subtraction isolates the interference term: $\delta I(t)=I_{\text{total}}(t)-\langle I_{R}\rangle$; here, $\langle I_{R}\rangle =E_{R}^{2}$.

Assuming the detected field fluctuations are Gaussian in the complex plane then ⟨Re(δI(t))Re(δI(t+τ))⟩=⟨Im(δI(t))Im(δI(t+τ))⟩). Then, the autocorrelation of δI(t) is

G2,iDWS(τ)≡⟨δI(t)δI(t+τ)⟩=2Re{⟨[ES(t)ei(ωt+ϕs(t))ERei(ωt+ϕR)*]×[ES(t+τ)ei(ω(t+τ)+ϕs(t+τ))ERei(ω(t+τ)+ϕR)*]*⟩}. (5)

Notice, $G_{2,\mathrm{iDWS}}(\tau )$ is proportional to the temporal autocorrelation function of the sample electric field. Specifically, upon carrying through the complex conjugation, simplifying, and expanding the product, we obtain

G2,iDWS(τ)=2⟨IR⟩Re{⟨ES(t)ES(t+τ)ei(ϕs(t)−ϕs(t+τ))⟩}=2⟨IR⟩G1(τ). (6)

Recall, this derivation assumes detection of a single sample field speckle, a spatially coherent reference field, as well as co-collimated and linearly co-polarized sample and reference fields. In practice, $G_{2,\mathrm{iDWS}}(\tau )=2\beta_{\mathrm{iDWS}}\langle I_{R}\rangle G_{1}(\tau )$, where $\beta_{\mathrm{iDWS}}$ depends on the mutual coherence of the sample and reference fields. A more in-depth discussion of the theory underlying iDWS can be found in the Supplemental Material of Ref. 53. Using the recovered interferometric, mean subtracted intensity autocorrelation function that is proportional to $G_{1}(\tau )$, we can perform nonlinear regression fitting based on the measured signal and a theoretical solution to the CDE to obtain the sample blood flow index, $BF_{i}$.

The SCOS approach is ultimately based on the same correlation diffusion equation given in Eq. (1). However, the experimental approach and its connection to theory are different from the DCS and iDWS techniques. SCOS utilizes slower camera–based detectors (CMOS or CCD cameras) to achieve highly parallelized detection of millions of speckles. SCOS information relies on speckle contrast, $\kappa (T_{\mathrm{Exp}})$, which is defined as the standard deviation of the measured intensity divided by its mean (either spatially or temporally). Here, $T_{\mathrm{Exp}}$ is the exposure time setting of the camera, which typically varies from tens of microseconds to tens of milliseconds. To estimate $BF_{i}$, the speckle contrast is related back to the CDE through an integration of the normalized electric field autocorrelation function, $g_{1}(\tau )$, via^57^

$$\kappa^{2}=\frac{2\beta_{scos}}{T_{\mathrm{Exp}}}\int_{0}^{T_{\mathrm{Exp}}}|g_{1}(\tau ){|}^{2}(1-\frac{\tau }{T_{\mathrm{Exp}}})d\tau .$$ (7)

Multiple noise source terms (e.g., shot noise and read noise) must be considered part of the SCOS estimation of $BF_{i}$ (see Sec. 2.2.3).^52^^,^^58^^,^^64^ For SCOS, the gains of using the exposure time integrated signals of many speckles are partially offset by the fact that the full temporal autocorrelation functions are not measured. In addition, the SCOS coherence parameter $\beta_{scos}$, which is related to the polarization state, spatial sampling, and source coherence, is not easily recoverable. Prior works have successfully determined $\beta_{scos}$ through acquisition of speckle contrast at multiple camera exposure times;^58^^,^^65^ however, adaptation of these methods has not been widespread. Thus, absolute estimation of $BF_{i}$ (or $\alpha D_{B}$) with SCOS is not typically reported. To avoid this issue, like most previous studies using SCOS,^52^^,^^66^ here we focus only on relative changes in flow measured by SCOS.

### 2.2 Instrumentation and Data Analysis

**Fig. 1:**
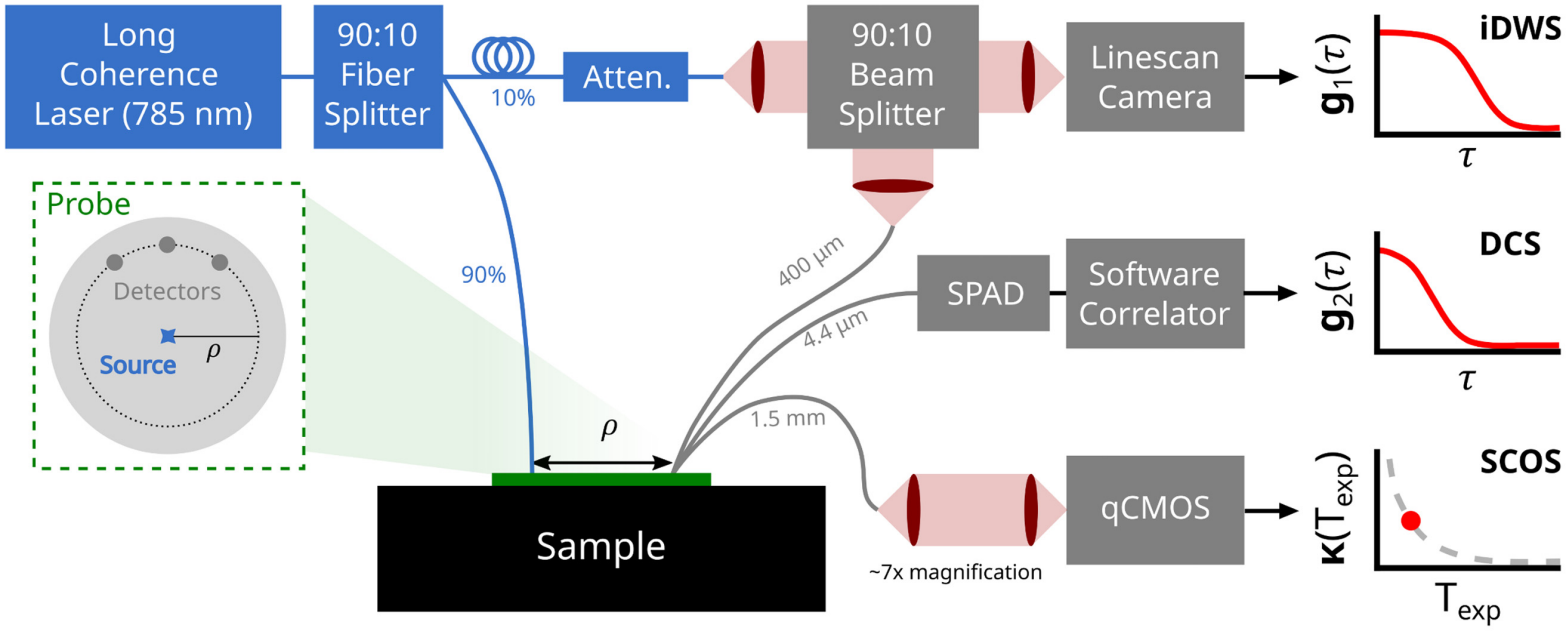
Schematic of the experimental setup used to perform simultaneous measurements with iDWS, DCS, and SCOS. All techniques shared the same single-mode fiber-coupled laser source. Each system used a separate optical fiber for light detection at ρ=2.5  cm from the source (fiber diameters shown). The center-to-center distances between adjacent detection fibers was 5 mm for *in vivo* measurements and 10 mm for *in vitro* measurements. iDWS and DCS measured the normalized electric field ($g_{1}(\tau )$) and intensity ($g_{2}(\tau )$ autocorrelation functions, respectively, at multiple delay times (τ). SCOS measured the speckle contrast (κ) at varying exposure times (here, we only show results for $T_{\exp}=1\;\text{m}s$ for both the *in vitro* and *in vivo* experiments).

A schematic of our experimental setup is shown in Fig. 1. Briefly, we designed the experiment such that the DCS, SCOS, and iDWS systems shared the same long coherence length source laser emitting at 785 nm (DL785-300-SO, Crystalaser, Reno, Nevada, United States). The laser consists of a single-stage optical isolator to attenuate possible back reflections, as well as coupling optics to deliver the beam into a single-mode optical fiber (780HP, Newton, New Jersey, United States) with an angle-polished connector. The use of a single-mode fiber instead of a multimode fiber facilitates a single-mode reference arm of the iDWS system and reduces sensitivity to environmental vibration/motion. Note, for DCS and SCOS, multimode fibers would suffice for source coupling, and recently a multimode source solution has also been proposed for iDWS.^67^ Separate optical fibers were used for DCS, SCOS, and iDWS detection; the fibers were positioned next to each other at exactly a 2.5 cm source-detector separation (SDS) (Fig. 1). Specifics of the DCS, iDWS, and SCOS instrumentation are explained in Secs. 2.2.1–2.2.3.

#### 2.2.1 DCS

The DCS system consists of 2 SPAD modules (SPCM-AQ4C, Excelitas, Exton, Pennsylvania, United States), each containing four individual detectors. We used a software autocorrelator to calculate $g_{2}(\tau )$ in real time with a 20 Hz sampling rate.^42^ Light collection was performed using a bundle of four single-mode fibers (780HP, Thorlabs, Newton, New Jersey, United States), each with a core diameter of 4.4  μm and a numerical aperture (NA) of 0.13. To improve DCS SNR, all four of these fibers are co-located within a single 2.5 mm stainless steel ferrule. This permits averaging of individual $g_{2}(\tau )$ functions recovered by each of the four SPADs. We directly recovered $BF_{i}$ (or $\alpha D_{B}$, presented in units of ${\mathrm{cm}}^{2}/s$) at each time point by fitting the $g_{2}(\tau )$ measurement to the semi-infinite solution of the CDE [Eq. (2), see Sec. 2.1]. Note, we assumed that the coherence parameter, $\beta_{\mathrm{DCS}}$, remained constant over time. We estimated it once for each experiment from fitting the mean $g_{2}(\tau )$ curve averaged across the first ∼1  min of data to the semi-infinite solution of the CDE for both $\beta_{\mathrm{DCS}}$ and $BF_{i}$.

#### 2.2.2 iDWS

The iDWS system is based on the Mach–Zehnder interferometer design. A fiber splitter (TW850R2A1, Thorlabs, Newton, New Jersey, United States) was used to deliver 10% of the source power to the reference arm and the remaining 90% to the sample. The 10% delivered into the reference arm underwent additional attenuation (BB-500-11-850-5/125-S-50-3A3A-1-1-ND-LL, OZ Optics, Carp, Ontario, Canada) and then was collimated (C430TME-B) and sent through a Powell lens (#43-473, Edmund Optics, Barrington, New Jersey, United States). Attenuation prevents camera saturation, and the Powell lens provides a fan beam with a near flat intensity profile to ensure uniform illumination on the line scan camera (spL4096-140 km, Basler, Exton, Pennsylvania, United States). The fan beam exiting the Powell lens was sent through an adjustable horizontal slit (VA100C, Thorlabs, Newton, New Jersey, United States), which acted as an aperture stop, and passed into the 10% input of a 90:10 beam splitter cube (BS026, Thorlabs, Newton, New Jersey, United States). Light emerging from the sample was collected with a 0.22 NA 400  μm core diameter multimode fiber, collimated (A397TM-B) and passed into the 90% input of the beam splitter cube. The co-collimated reference and sample light emerging from the beam splitter cube were focused via a cylindrical lens (AYL3026-B, Thorlabs, Newton, New Jersey, United States) onto the line scan camera, i.e., the light was focused to uniformly illuminate the 512×2  pixels used in the camera. We operated the camera at a line rate of 333 kHz to obtain intensity data every 3  μs from the central 512×2  pixels (W × H), which are then vertically binned, i.e., the two rows of 512 pixels are summed pixelwise. The optical setup used for iDWS provided a spatial autocorrelation half-width at half maximum of ∼1.5 horizontal pixels; we estimated this ratio using the half-width half-maximum of the Gaussian fit to the spatial autocorrelation of the measured interferometric intensity sequence acquired in an intralipid phantom at 5 mm source-detector separation.^68^ During measurements, the raw continuous pixel data from the line scan camera are concatenated and saved as 512×3334 images (i.e., each frame contains 3334 time points).

The raw iDWS camera intensity data were processed to obtain interferometric intensity autocorrelation functions using custom MATLAB (MathWorks, Natick, Massachusetts, United States) software (recall, for the iDWS measurement, the interferometric intensity autocorrelation function ($G_{2,\mathrm{iDWS}}(\tau )$) is directly proportional to the real part of the electric field autocorrelation function). Specifically, the saved intensity images were loaded into MATLAB and concatenated to form a single large array. To isolate the interference term, the concatenated array undergoes a 0.1 s rolling mean subtraction to obtain δI(x,t), where x corresponds to a vertically binned pixel’s horizontal spatial location within the line scan camera. To account for correlation due to neighboring pixels sampling the same speckle, we performed an additional spatial filtering step at every time point across the vertically binned 512 pixels. Specifically, δI(x,t) was convolved across x with a Gaussian window of peak value 1 and standard deviation 0.9669. For additional information on determining this optimal spatial binning filter, the interested reader is directed to Ref. 69. This mean subtracted and binned raw intensity data were then used to obtain the temporal intensity autocorrelation functions via a fast Fourier transform approach at a 20 Hz sampling rate (0.05 s integration time) using the standard xcorr function in MATLAB.^70^ Briefly, via the Wiener–Khinchin theorem, the temporal intensity autocorrelation function is $G_{2,\mathrm{iDWS}}(x,\tau )=F^{-1}\{|F\{\delta I(x,t)\}{|}^{2}\}$, where F and $F^{-1}$ refer to the forward and inverse Fourier transform, respectively, and δI(x,t) is the mean subtracted, temporal intensity of one of the binned 512 pixels acquired in parallel by the line scan camera. Via this approach, we obtain $G_{2,\;\mathrm{iDWS}}(x,\tau )$ for each pixel over 4000 uniformly spaced τ (3  μs spacing).

The individual autocorrelation functions of the spatially and vertically binned 512 camera pixels were then summed together to improve the signal-to-noise ratio. To account for any additional decay contributed by the reference arm signal and sample back reflections, an additional 10 s measurement was performed before each session with the sample arm blocked (i.e., the source fiber was removed from the probe; see Fig. 1). The autocorrelation of the reference arm intensity (obtained using the Wiener–Khinchin theorem) was then subtracted from the autocorrelation functions collected with sample illumination. The square of the reference-corrected autocorrelation functions was then fit by minimizing the following cost function:

$$\text{cost}Fc=\underset{\tau }{\sum }(G_{2,\mathrm{iDWS}}^{2}-(A*g_{1}(\tau ){)}^{2}{)}^{2},$$ (8)

where A compensates for the system’s unknown coupling coefficient, and $g_{1}(\tau )$ is the theoretical normalized electric field autocorrelation function [Eq. (2)]. The sum over τ was performed up to the first delay time for which $g_{1}(\tau )<0$. Please note, here we are fitting $G_{2,\mathrm{iDWS}}^{2}(\tau )$ as opposed to $G_{2,\mathrm{iDWS}}(\tau )$ as this approach proved more robust against noise (see Fig. 9 in Appendix). For iDWS, we used the semi-infinite solution of the CDE [Eq. (2)] to recover $BF_{i}$ (or $\alpha D_{B}$) and A for each collected frame. (Note, in contrast to the single parameter fitting from DCS, we are fitting for two parameters with iDWS).

#### 2.2.3 SCOS

The SCOS system imaged the tip of a 1.5 mm diameter, 0.5 NA, step-index detector fiber (FT1500URT) with a CMOS camera (C15550-20UP, Hamamatsu, Hamamatsu City, Japan) operating at a rate of 120 frames per second. To ensure adequate spatial sampling of the acquired speckle pattern,^71^ we used a custom lens assembly providing ∼7× magnification to obtain a speckle-to-pixel size ratio of ∼2.3. Each camera frame was first cropped to only include pixels forming the image of the fiber tip (totaling $∼3\times 10^{6}\;\text{pixels}$). Then, one speckle contrast, $\kappa (T_{\mathrm{Exp}})$, was computed for each image using the method proposed by Kim et al.^52^ Their method, which builds upon previous speckle contrast noise correction models,^58^^,^^64^ corrects for multiple noise contributions, including a dark offset, as well as shot, read, and quantization noise. It also corrects for spatial variations in the speckle image that are not attributable to the sample.

Briefly, the measured speckle contrast, $\kappa_{\mathrm{Meas}}(T_{\mathrm{Exp}})$ was calculated for each 7×7 nonoverlapping window after removing a dark count offset from each window; speckle contrast is defined as the spatial standard deviation (σ) divided by the spatial mean (μ) of the dark-corrected intensities across each window. For the dark offset correction, we collected the mean camera counts during a 5 s period when no source light was delivered to the sample.

To estimate $BF_{i}$ with SCOS, multiple noise terms are subtracted from the measured contrast, $\kappa_{\mathrm{Meas}}(T_{\mathrm{Exp}})$, for each window. The fundamental speckle contrast, $\kappa_{f}$, used for estimating $BF_{i}$ can be defined as

$$\kappa_{f}^{2}=\kappa_{\mathrm{Meas}}^{2}-\kappa_{\text{Read}}^{2}-\kappa_{\text{Shot}}^{2}-\kappa_{\text{Quant}}^{2}-\kappa_{\text{Spatial}}^{2},$$ (9)

where $\kappa_{\text{read}}$ is the read noise contribution, which was estimated from the speckle contrast of the images collected with no source light (i.e., $\kappa_{\text{Read}}^{2}=\sigma_{\text{Dark}}^{2}/\mu^{2}$), $\kappa_{\text{Shot}}^{2}=g/\mu$ is the shot noise contribution (g=9.64 is the measured camera gain^66^), and $\kappa_{\text{quant}}^{2}=1/(12\mu^{2})$ is the quantization noise. (Note, accurate estimations of g are essential for obtaining accurate SCOS measurements;^66^ see Fig. 10 in the Appendix).

**Fig. 2:**
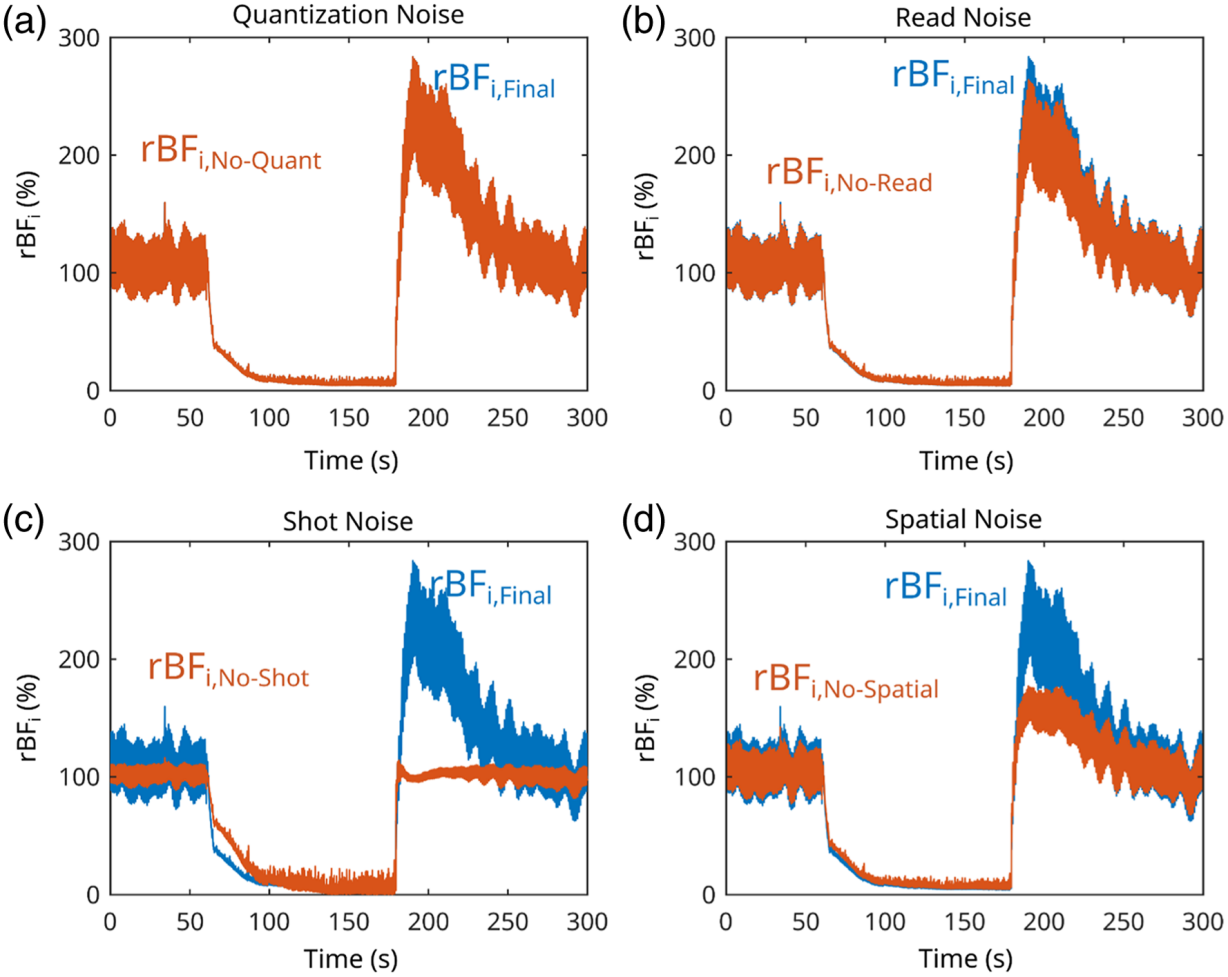
Example of the effects of specific noise correction on the recovered speckle contrast. The blue line in each plot represents the final corrected value of $rBF_{i}$ and the orange line represents the $rBF_{i}$ recovered if a specific source of noise is ignored. The sources of noise ignored were (a) quantization noise, (b) read noise, (c) shot noise, and (d) spatial noise.

The spatial speckle noise correction is calculated from $\kappa_{\text{Spatial}}^{2}=\sigma_{\text{Spatial}}^{2}/\mu^{2}$, where $\sigma_{\text{Spatial}}^{2}=\sigma_{\mathrm{Avg}}^{2}-g*\mu_{\mathrm{avg}}/N_{\mathrm{avg}}$ is the shot noise corrected spatial variance obtained by temporally averaging $N_{\mathrm{Avg}}=100$ camera frames;^52^ $\sigma_{\mathrm{Avg}}$ and $\mu_{\mathrm{Avg}}$ are, respectively, the spatial standard deviation and mean of the temporally averaged camera frames. (Note, $\sigma_{\text{Spatial}}^{2}$ is calculated once for each set of $N_{\mathrm{Avg}}$ frames, and it is assumed to be constant across the frames used to calculate it). Figure 2 shows an example of how the SCOS noise corrections affect the final recovered value of the relative changes in the flow index ($rBF_{i}$) in an exemplary arm cuff occlusion experiment (see Sec. 2.3 for more details). As seen in Fig. 2, shot and spatial noise corrections are the most critical to the recovery of relative blood flow, followed by read noise, and finally quantization noise which was essentially negligible.

After performing the necessary noise corrections, we recovered $BF_{i}$ from fitting the average $\kappa_{f}^{2}$ measurement (i.e., the mean $\kappa_{f}^{2}$ across all 7×7 nonoverlapping windows) at one camera exposure time ($T_{\text{Exp}}$) to Eq. (4), wherein we used the normalized semi-infinite solution of the CDE for $g_{1}(\tau )$, i.e., $g_{1}(\tau )=G_{1}(\tau )/G_{1}(0)$ with $G_{1}(\tau )$ given by Eq. (2). As it is experimentally challenging to estimate $\beta_{SCOS}$, we opted to re-scale the SCOS measurements; for the *in vitro* experiment (see Sec. 2.3.1), we matched the recovered values to the expected Brownian diffusion coefficient based on the Stokes–Einstein’s relation at 21°C to 22°C, and for the *in vivo* experiments (see Sec. 2.3.2), we only report changes in $BF_{i}$ from the 1 min baseline period (i.e., $rBF_{i}$). In addition, for the *in vitro* and *in vivo* measurements reported herein, we set the camera exposure time ($T_{\mathrm{Exp}}$) to 1  ms. (Note, for the *in vitro* experiment, we collected SCOS data with $T_{\text{exp}}=0.25$, 0.5, 1, 2, 4 and 8  ms, but we only report the results for 1  ms exposure time as there was little difference among $BF_{i}$ values recovered using different exposure times).

### 2.3 Experimental Data

#### 2.3.1 *In vitro*: microsphere phantom

To investigate the accuracy and precision of DCS, iDWS, and SCOS, we used a polystyrene microsphere phantom with particles of 195 nm diameter (Bang Laboratories, Fishers, Indiana, United States)^72^ suspended in water (0.29% volume fraction of microspheres), with water-soluble nigrosine ink added for absorption. The phantom volume of 0.5 L was inside a 9×9×8  cm (length ×width × depth) 3D printed container made of black PETG. According to Stokes–Einstein’s relation, the theoretical particle Brownian diffusion coefficient, $D_{b,\mathrm{Theo}}$, depends on microsphere size, water temperature, and water viscosity.^73^ Specifically, $D_{b,\mathrm{Theo}}$ is

$$D_{b,\mathrm{Theo}}=\frac{k_{B}T}{6\pi a\eta },$$ (10)

where $k_{B}$ is Boltzmann’s constant (i.e., $1.380649^{-23}\;\text{Joules}/\text{Kelvin}$), T is suspension temperature in degrees Kelvin, and a is the microsphere radius in meters. η=η(T) is the temperature-dependent viscosity of water in units of Pascal × seconds, which can be estimated from Vogel’s equation^73^

$$\eta (T)=10^{-3}\times \exp(-3.7188+(\frac{578.919}{-137.546+T})).$$ (11)

To obtain a range of $D_{b,\mathrm{Theo}}$ values for comparison, we varied the phantom temperature from ∼7°C to 22°C during the DCS, iDWS, and SCOS measurements. To this end, the phantom was kept overnight in a conventional fridge (∼5°C). On the measurement day, after performing all the necessary iDWS and SCOS calibration steps (see Secs. 2.2.2 and 2.2.3), the phantom was moved to a low-temperature oven (set to ∼50°C), and DCS, iDWS, and SCOS measurements were performed in the semi-infinite geometry (i.e., with probe mounted flush to the phantom surface and centered), as depicted in Fig. 1, for ∼1  h. A low-temperature oven was used to reduce the total measurement time without introducing temperature gradients on the phantom. We continuously measured the phantom temperature with two temperature sensors (DS18B20, Adafruit Industries LLC, New York City, New York, United States) positioned at opposite corners and varying depth (∼2 and ∼6  cm below the surface). Temperatures from the two sensors were not significantly different, suggesting no temperature gradients on the phantom; the mean temperature across both sensors was then used to estimate $D_{b,\mathrm{Theo}}$ from Eqs. (10) and (11) at each time point. Note, as the changes in $D_{b,\mathrm{Theo}}$ due to increasing temperature occur slowly, we restricted the SCOS and iDWS sampling times to avoid data pile-up. Specifically, we collected 100 SCOS frames and 200 iDWS frames every 2 min at sampling rates of 120 and 20 Hz, respectively. DCS, by contrast, was sampled continuously throughout the experiment at a rate of 20 Hz.

Finally, to accurately estimate the particle diffusion coefficient ($D_{b}$) with DCS, iDWS, or SCOS, knowledge of the phantom’s optical properties is important. Accordingly, we used a separate, commercial frequency-domain diffuse optical spectroscopy (FD-DOS) system (ImageNet, ISS Medical, Champaign, Illinois, United States) to measure the phantom’s $\mu_{a}$ and $\mu_{s}^{\prime }$ at room temperature. FD-DOS measurements were performed at three wavelengths (727, 750, and 827 nm) and 8 SDS (0.7 to 3 cm). We used the multidistance semi-infinite solution of the frequency-domain photon diffusion equation to recover the optical properties at each wavelength.^7^ Assuming a power law model for scattering (i.e., $\mu_{s}^{\prime }(\lambda )=A(\lambda /500\;\mathrm{nm}{)}^{-b}$) and a linear model for $\mu_{a}(\lambda )$, we extrapolated the optical properties to the 785 nm wavelength used in the flow experiment.^7^ Note, changes in optical properties versus temperature (over the range in our study) are expected to be negligible.^72^

#### 2.3.2 *In vivo*: forearm measurements

We also investigated differences that may arise when measuring blood flow in more clinically relevant forearm measurements. These *in vivo* human measurements enabled us to explore the agreement among each technique when measuring heterogeneous tissues. For this work, we performed a series of arm cuff occlusion experiments in healthy adult volunteers. All subjects provided informed written consent, and all experimental procedures were approved by the Institutional Review Board of the Children’s Hospital of Philadelphia.

**Fig. 3:**
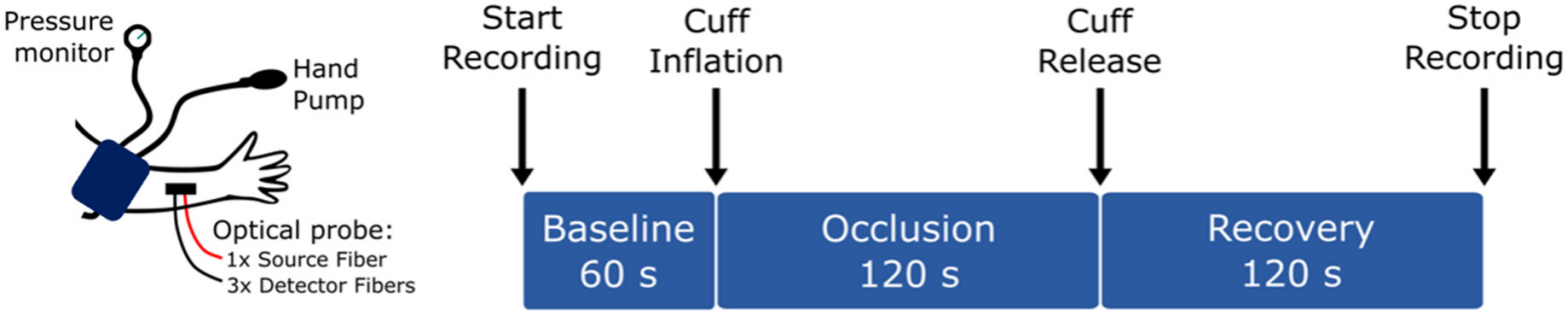
Experimental protocol for monitoring blood flow in the forearm before/during/after arm cuff occlusion in healthy adults. Flow was measured simultaneously with DCS, SCOS, and iDWS techniques; the source-detector separation for all three techniques was 2.5 cm. The protocol was repeated once after a rest period of at least 5 min.

Arm cuff occlusion experiments provide a predictable blood flow response with a large dynamic range.^6^^,^^52^^,^^58^ The optical probe (see Fig. 1) was placed on the proximal end of the dorsal aspect of the left forearm, and simultaneous DCS, iDWS, and SCOS measurements were performed before/during/after two sequential arm cuff occlusions (Fig. 3). Each occlusion protocol comprised a 1 min baseline, 2 min of arterial occlusion (induced by manual inflation of a blood pressure arm cuff placed around the bicep to ∼210  mmHg), and 2 min of recovery (i.e., to fully capture the post-occlusion hyperemic flow response); there was at least a 5 min rest period among occlusion protocols. The DCS, iDWS, and SCOS sampling rates were 20, 20, and 120 Hz, respectively, and the SCOS camera exposure time was 1  ms.

Note, we were unfortunately unable to independently measure the optical properties for each subject; we thus assumed $\mu_{a}=0.1\;{\mathrm{cm}}^{-1}$ and $\mu_{s}^{\prime }=6\;{\mathrm{cm}}^{-1}$ for all experimental time points. To avoid the cofound of errors in absolute $BF_{i}$ induced by errors in the assumed optical properties, here we only report the relative $BF_{i}$ normalized by the mean $BF_{i}$ across the baseline period (i.e., $rBF_{i}$).

In addition to probing how each modality responds to large changes in blood flow over seconds, we also leveraged the baseline periods to investigate each modality’s measurement of pulsatile fluctuations in blood flow during the cardiac cycle. Using data from the 1 min baseline period, we obtained an average pulsatile waveform from each technique. We used the SCOS $BF_{i}$ measurements (due to their higher SNR and higher sampling rate) as a reference to identify the timing of each systolic peak. To reduce low-frequency effects on the individual waveforms (e.g., from respiration cycles), we divided each heartbeat waveform by its mean, separately for each technique. This additional normalization step reduced the interquartile range of the final averaged waveforms. We then calculated the pulsatility index (PI) from each individual pulsatile waveform. PI is defined as the peak-to-peak amplitude of the waveform divided by its mean. Here, we report the mean PI calculated for each technique, after the removal of outliers from noisier waveforms; we used a standard MATLAB function for this purpose (i.e., *rmoutliers*).

### 2.4 Statistical and Qualitative Analysis

For the *in vitro* phantom experiment, we used linear regression and nonparametric Bland–Altman analyses to compare the $D_{b}$ values recovered by SCOS, iDWS and DCS to $D_{b,\mathrm{Theo}}$ [computed with Eqs. (10) and (11)]. Specifically, we evaluated the linear relationships, the recovery bias, limits of agreement (LOA) and the nonparametric reproducibility coefficient (RPC) of each technique. We also calculated the Pearson correlation coefficients among the $D_{b}$ values recovered with each technique (i.e., $r_{x-y}$, with x and y representing the techniques being compared). For the *in vivo* experiments, we performed a Friedman test to determine whether the PI at baseline and maximum post-occlusion $rBF_{i}$ (i.e., $rBF_{i,\max}$) differed across the three measurement techniques. If significance was found, post hoc pairwise comparisons among each technique were then conducted using Wilcoxon signed-rank tests. For the *in vivo* experiments, we also performed a pairwise correlation analysis separately comparing the $rBF_{i,\max}$ and PI values recovered from each technique. All statistical tests were two-sided, and p<0.05 was deemed significant. In addition to this more rigorous quantitative analysis, we also include data visually comparing the autocorrelation functions acquired from iDWS and DCS. To do this, we show examples of $g_{1}(\tau )$ recovered via iDWS with the corresponding $g_{1}(\tau )$ recovered via DCS; note, for DCS we used the $g_{2}(\tau )$ measurement and Siegert’s relation (i.e., $g_{1}(\tau )=\sqrt{|g_{2}(\tau )-1|/\beta_{\mathrm{DCS}}}$).

## 4 Results

### 4.1 *In vitro* Microsphere Phantoms

**Fig. 4:**
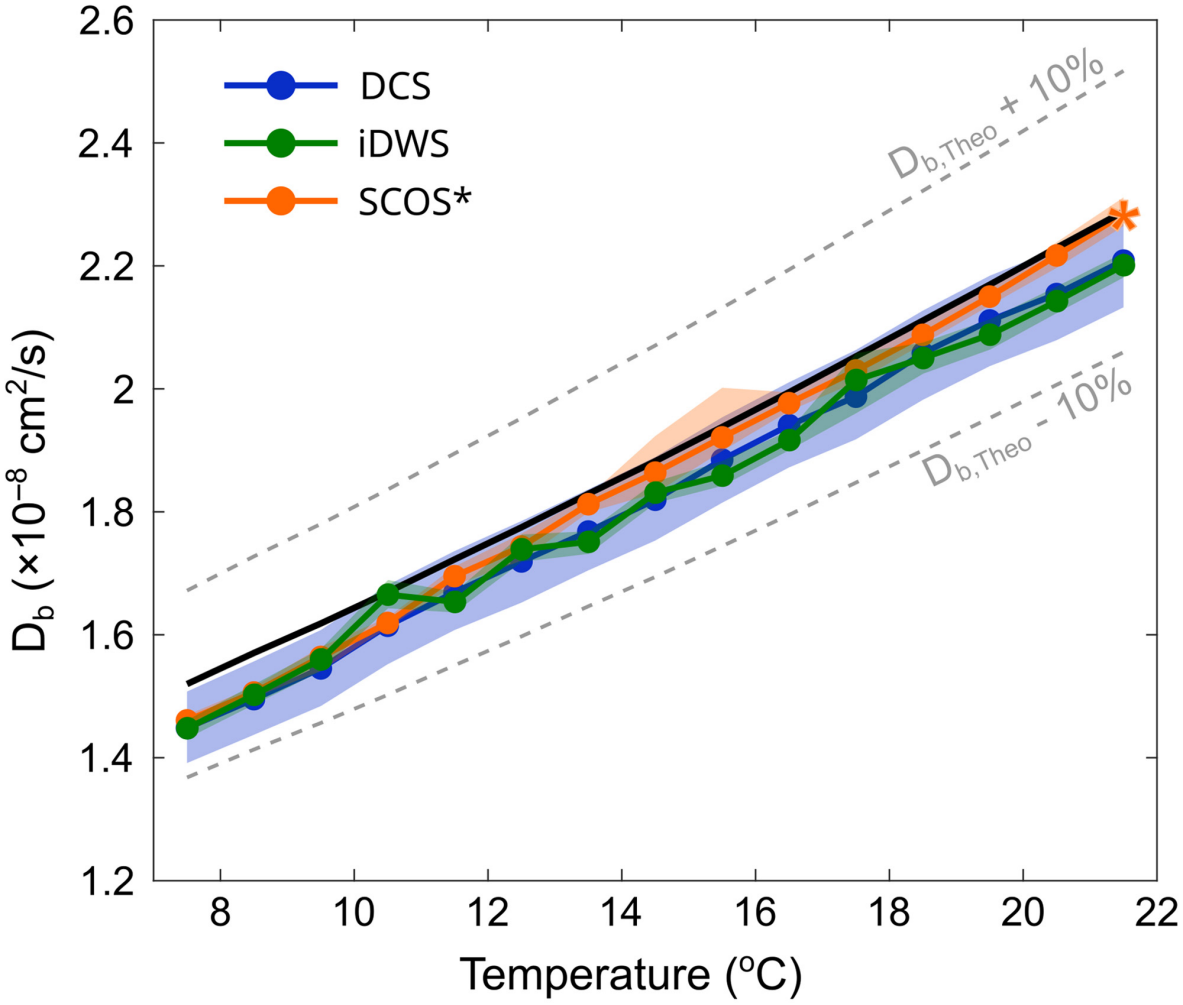
Measured $D_{b}$ plotted as a function of temperature and instrumental system for 195 nm microsphere particles with SDS of 2.5 cm. The solid black line represents the theoretical $D_{b}$ value obtained using Stokes–Einstein’s equation. For visualization purposes, we calculated the median (represented by circles) and interquartile range (represented by the shaded regions) of the DCS (blue), iDWS (green), and SCOS (orange) measurement, for each 1°C change (from 8°C to 22°C). $D_{b}$ values for DCS were obtained using the averaged $g_{2}(\tau )$ from four parallel detectors. *SCOS $D_{b}$ value is normalized to the theoretical value of $D_{b,\mathrm{Theo}}$ at 21°C-22°C (represented by the orange star).

To assess the accuracy and precision of each technique, we compared the theoretical estimate of $D_{b,\mathrm{Theo}}$ as a function of temperature to the values of $D_{b}$ recovered by DCS, iDWS, and SCOS in our *in vitro* phantom experiment. As a reminder, the recovered $D_{b,\mathrm{SCOS}}$ values for SCOS were normalized to the theoretical value from Stokes–Einstein’s relation during the last minute of data collection (represented by star in Fig. 4) and should only be interpreted as relative changes. Furthermore, recall that as DCS data were continuously acquired throughout the experiment, whereas SCOS and iDWS were only intermittently measured; there are accordingly more DCS measurements for comparison.

**Fig. 5:**
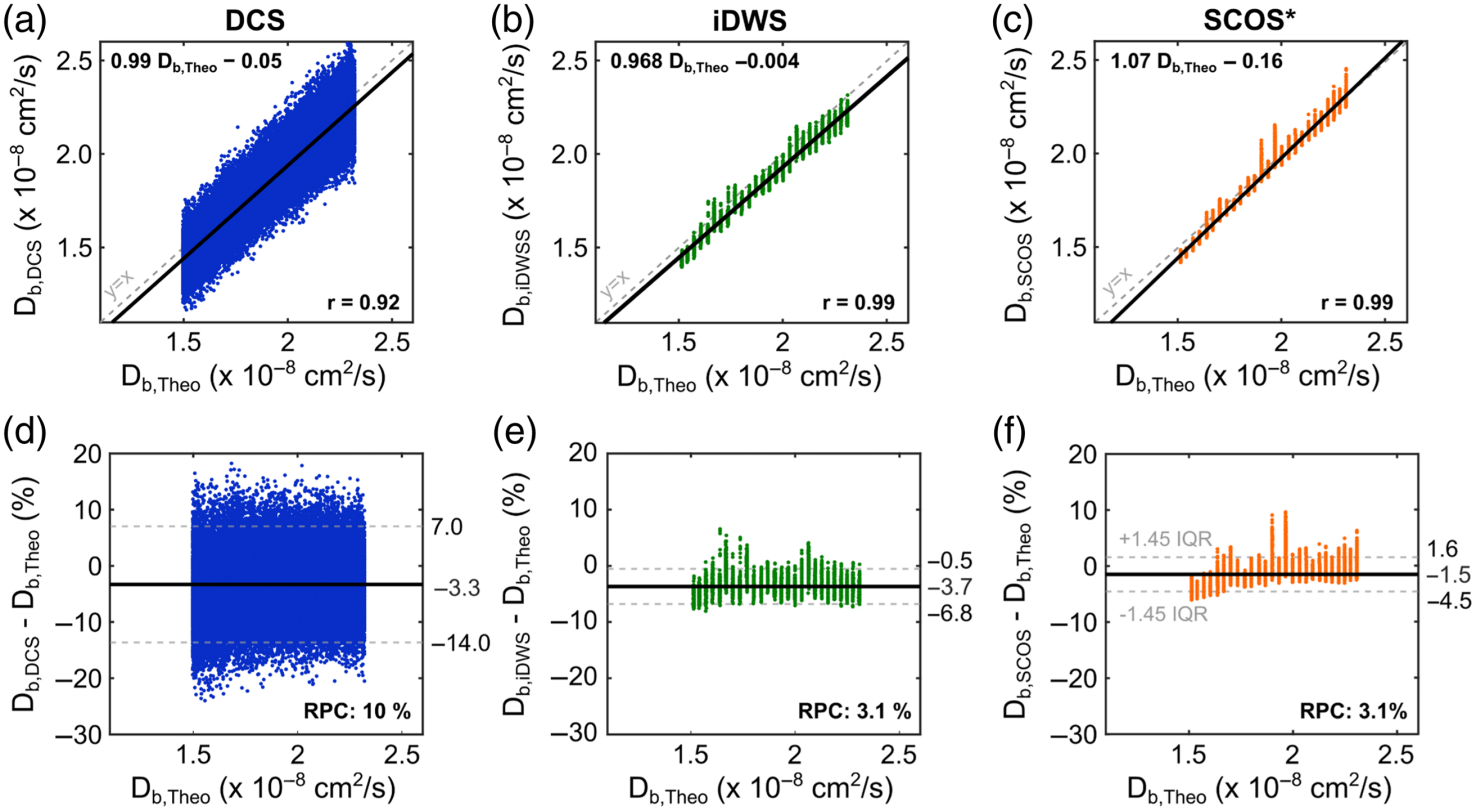
Bland–Altman analysis for our *in vitro* phantom experiment using a water suspension of polystyrene microspheres with 195 nm diameter. The first row shows the correlation analysis comparing the expected Brownian coefficients ($D_{b,\mathrm{Theo}}$) based on Eqs. (10) and (11) to the values measured with (a) DCS (blue dots, $D_{b,\mathrm{DCS}}$), (b) iDWS (green dots, $D_{b,\mathrm{iDWS}}$), and (c) SCOS (orange dots, $D_{b,\mathrm{SCOS}}$). The solid black lines represent the linear fits between $D_{b,\mathrm{Theo}}$ and the values recovered from each technique, whereas the dashed gray lines represent the unity line. The second row represents the Bland–Altman comparing (d) $D_{b,\mathrm{DCS}}$, (e) $D_{b,\mathrm{iDWS}}$, and (f) $D_{b,\mathrm{SCOS}}$ to the expected Brownian coefficients. The reproducibility coefficients (RPC) and the limits of agreement are shown for each modality. All measurements were acquired at 2.5 cm source-detector separation. DCS was continuously collected with 20 Hz sampling rate, whereas SCOS (120 Hz sampling) and iDWS (20 Hz) data were intermittently acquired; we specifically recorded 100 SCOS frames and 200 iDWS frames every 2 min. $D_{b}$ values for DCS were obtained using the averaged $g_{2}(\tau )$ from four parallel detectors. *All SCOS values were normalized to the theoretical value of $D_{b,\mathrm{Theo}}$ during the last minute of data collection.

First, as seen in Figs. 4 and 5, the $D_{b}$ measurements of all three techniques were strongly correlated with $D_{b,\mathrm{Theo}}$ values ($r_{\mathrm{DCS}-\mathrm{Theo}}=0.92$, $r_{\mathrm{iDWS}-\mathrm{Theo}}=0.99$, and $r_{\mathrm{SCOS}-\mathrm{Theo}}=0.99$). Linear regression analyses of $D_{b}$ and $D_{b,\mathrm{Theo}}$ are shown in Figs. 5(a)–5(c) (slopes and intercepts are reported in the figure). Interestingly, the linear relationship between $D_{b}$ recovered by SCOS and the theoretical $D_{b,\mathrm{Theo}}$ values appeared to deviate from the unity line for lower temperatures. This may be explained by issues with source laser stability and environmental effects from vibration of the heating ventilation and air conditioning system. Laser instabilities can lead to a reduction of $\beta_{\mathrm{SCOS}}$, and a change in laser power output can directly impact the calculation of speckle contrast (κ=σ/μ). In addition, the large and relatively rigid solid core fiber used by SCOS is especially inept at absorbing these vibrations, which can affect the mode distribution resulting in the addition of speckle variation at the camera that is not attributable to the sample of interest.

In addition to the effects of source instability on SCOS, there is also an effect on iDWS. The values of $D_{b}$ recovered from iDWS during the microsphere experiment display occasional irregularities. Mode-hopping of the source laser will result in both unwanted coherence gating of photon time-of-flights, as well as variations in the reference arm intensity, which is assumed to be constant in all analysis. That said, the degradation of measured values was small, and pairwise correlation analysis revealed highly correlated $D_{b}$ values across modalities (as expected from Figs. 4 and 5); we specifically found $r_{\mathrm{DCS}-\mathrm{iDWS}}=0.92$, $r_{\mathrm{DCS}-\mathrm{SCOS}}=0.92$, and $r_{\mathrm{iDWS}-\mathrm{SCOS}}=0.98$. When comparing the absolute $D_{b}$ recovered from both DCS and iDWS, we observed a similar significant negative bias for both techniques (−3.3% and −3.7%, respectively, for DCS and iDWS; p<0.001). This difference can likely be attributed to errors in the recovery of the optical properties or small errors in the measured temperature. With FD-DOS, the recovered absorption and reduced scattering coefficients were, respectively, $\mu_{a}=0.104\pm 0.002\;{\mathrm{cm}}^{-1}$ and $\mu_{s}^{\prime }=6.30\pm 0.09\;{\mathrm{cm}}^{-1}$. 

**Fig. 6:**
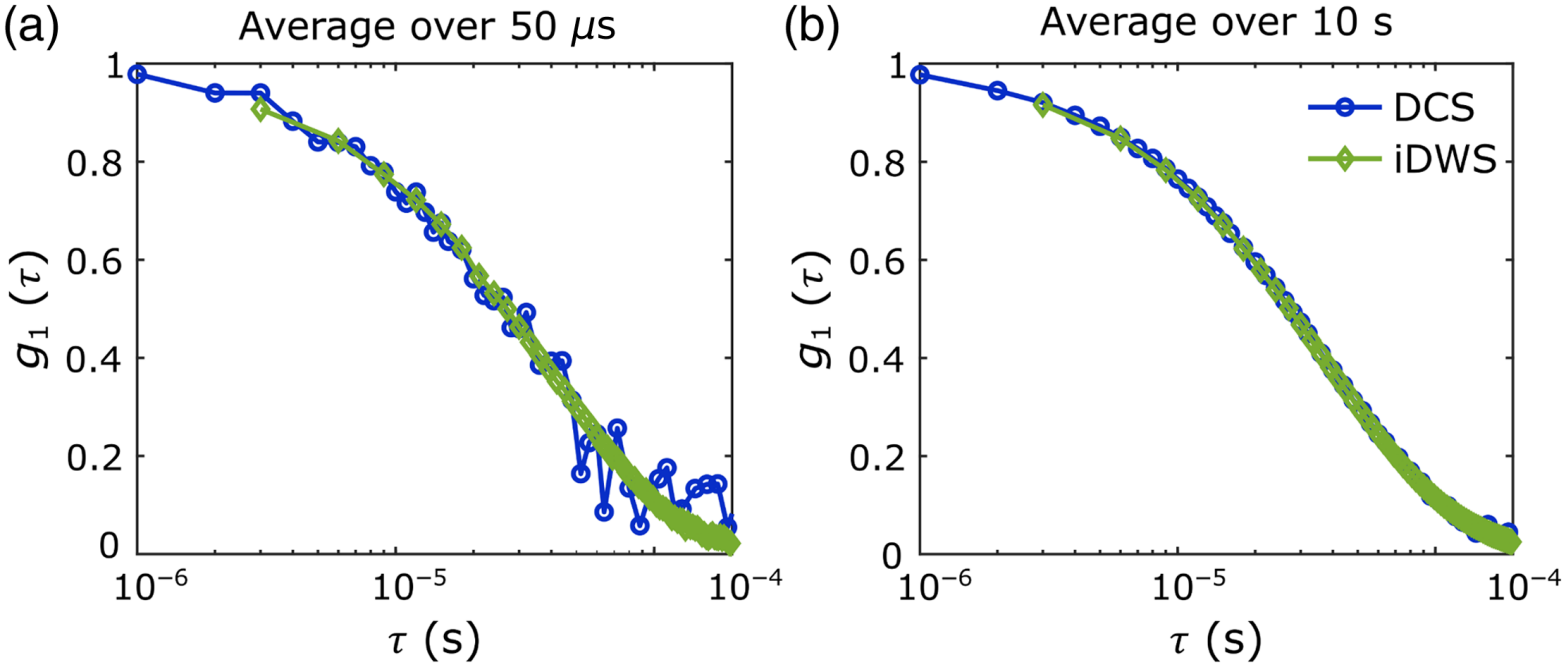
Comparison of the electric field autocorrelation functions (i.e., $g_{1}(\tau )$) recovered with iDWS (green curves) and DCS (blue curves) for the microsphere phantom experiment with 195 nm microspheres for (a) a single $g_{1}(\tau )$ collected with 50  μs integration time and (b) the measured $g_{1}(\tau )$ with a 10 s averaging. The data were recovered from the first 10 s of the phantom experiment. DCS curves were obtained by averaging $g_{2}(\tau )$ obtained with four parallel detectors and applying Siegert’s relation (i.e., $g_{1}(\tau )=\sqrt{|g_{2}(\tau )-1|/\beta_{\mathrm{DCS}}}$).

Importantly, although we observed a bias in the absolute recovery of $D_{b,\mathrm{Theo}}$, the DCS and iDWS measurements were in excellent agreement with each other (as seen by the similar recovery bias). As seen in Fig. 6, both techniques recovered nearly identical electric field autocorrelation functions. We remind the reader that for DCS, $g_{1}(\tau )$ was calculated using Siegert’s relation (i.e., $g_{1}(\tau )=\sqrt{|g_{2}(\tau )-1|/\beta_{\mathrm{DCS}}}$).

Finally, we also compared the precision of each technique in our *in vitro* phantom measurements [Figs. 5(d)–5(f)]. DCS showed the largest dispersion (LOA, –14% to 7%; RPC, 10%), followed by iDWS (LOA, –6.8% to –0.5%; RPC, 3.1%) and SCOS (LOA, –4.5% to 1.6%; RPC, 3.1%). Importantly, SCOS obtained a similar spread (and thus a similar signal-to-noise ratio) to iDWS with a 6× faster sampling rate. As a reminder, SCOS was collected at 120 Hz, whereas both DCS and iDWS were collected at 20 Hz.

### 4.2 *In Vivo* Forearm Measurements

**Fig. 7:**
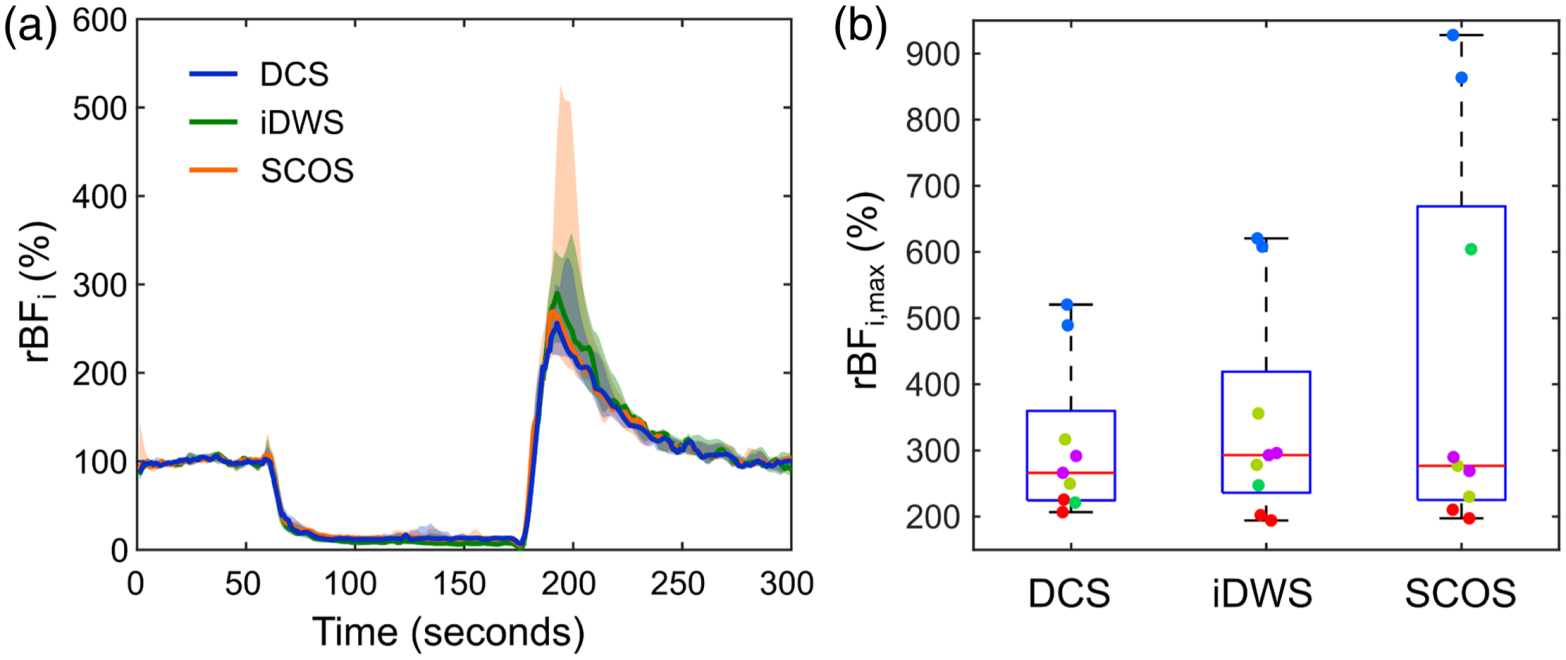
(a) Temporal measurements of relative flow for each modality averaged across all arm cuff occlusion protocols (i.e., 10 protocols obtained from measuring each of the five subjects twice). Shaded regions provide the interquartile range for their respective modality. (b) Distribution of relative blood flow indices recovered by each modality at the peak hyperemic response. Each color represents a different subject.

Two arm cuff experiments were performed in N=5 subjects [3 males, 2 females; mean age 34.4 years (range 22 to 58); 4 white, 1 Asian] for a total of 10 experimental runs. In one of the 10 *in vivo* forearm experiments, we observed $\kappa_{f}^{2}<0$ during the post-occlusion period, representing a breakdown of the SCOS noise correction model. To avoid the comparison of unrealistic flows from SCOS, we opted to exclude this run from the average response analysis. Figure 7(a) presents the average DCS, iDWS, and SCOS measurements of relative blood flow index ($rBF_{i}$) across the remaining nine arm cuff occlusion runs. The average flow changes during the baseline, and occlusion and recovery periods were in good agreement across all modalities.

When comparing hyperemic peak values ($rBF_{i,\max}$) across all modalities, no statistically significant differences were found [p=0.6, Fig. 7(b)]; the $rBF_{i,\max}$ values from each technique were highly correlated (i.e., $r_{\mathrm{DCS}-\mathrm{iDWS}}=0.99$, $r_{\mathrm{DCS}-\mathrm{SCOS}}=0.84$, and $r_{\mathrm{SCOS}-\mathrm{iDWS}}=0.87$). Finally, when comparing the absolute $BF_{i}$ values recovered with DCS and iDWS at baseline, we found that the values were highly correlated (r=0.94), with a linear relationship close to the unity line (i.e., $BF_{i,i\mathrm{DWS}}=a*BF_{i,\mathrm{DCS}}+b$, with a=0.97±0.11 and $b=(0.03\pm 0.08)\times 10^{-8}\;{\mathrm{cm}}^{2}/s$). (As a reminder, we were unable to estimate an absolute $BF_{i}$ with SCOS, so this comparison was limited to DCS and iDWS).

**Fig. 8:**
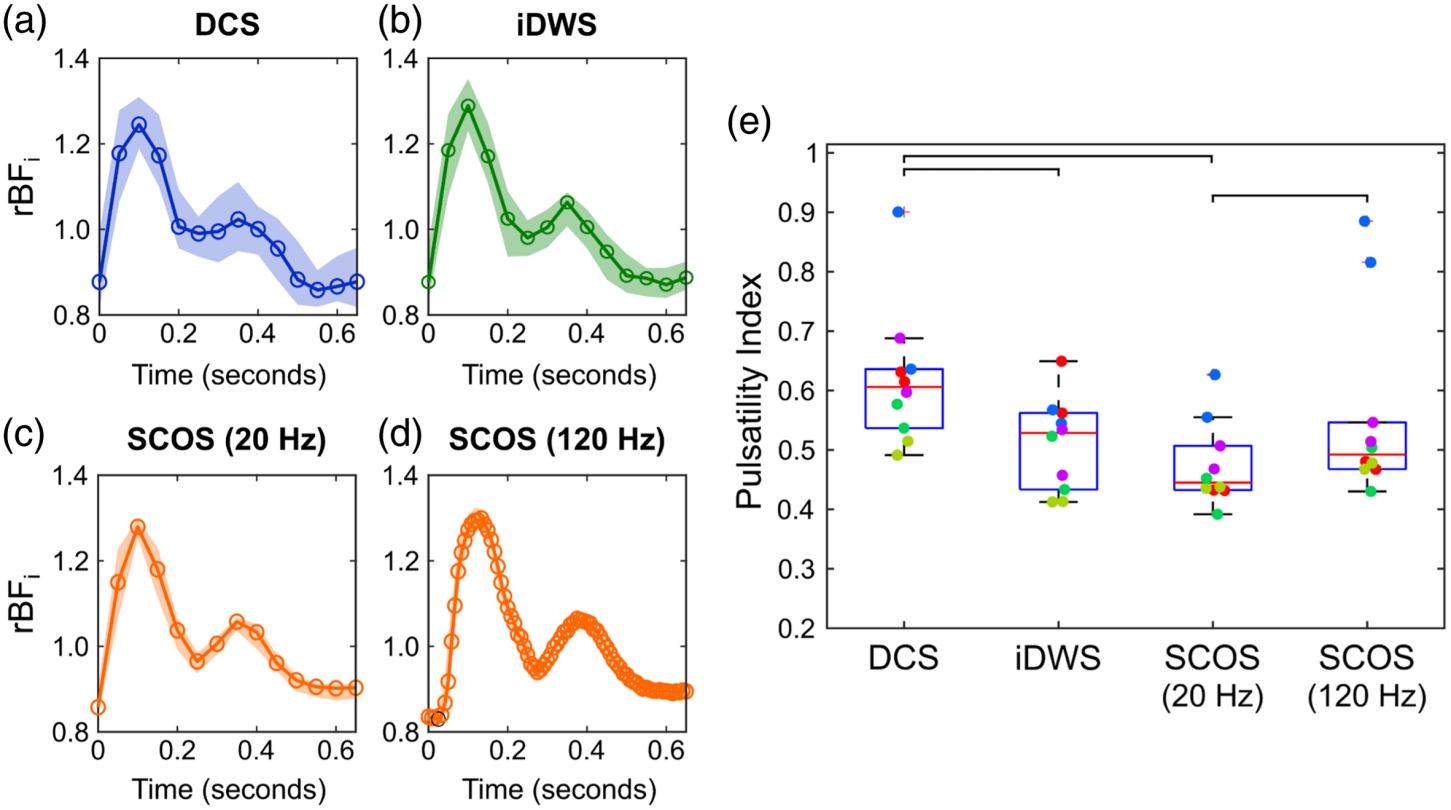
Blood flow pulsatility waveforms in a single subject recovered by averaging across all individual pulses during the baseline period measured with (a) DCS, (b) iDWS, (c) SCOS at 20 Hz, and (d) SCOS at 120 Hz. Note, SCOS at 120 Hz was used to synchronize all waveforms by identifying systolic times for each individual pulse. (e) Box plots of DCS, iDWS, and SCOS measurements of the mean pulsatility index across each baseline period in each subject (n=5 subjects, two baseline periods per subject, as we performed two cuff-occlusion protocols per subject). The dots superimposed on the boxplots are the individual measurements; each color represents a different subject. The star (*) represents a significant (p<0.05) post hoc pair-wise Wilcoxon signed-rank test.

We also examined pulsatile changes in flow during the cardiac cycle obtained with each modality. Figures 8(a)–8(d) show the normalized pulsatile flow signals averaged across all heartbeats during the baseline period of a single subject (the flow waveform for each heartbeat was normalized by its mean). Here, we opted to present SCOS both at its native 120 Hz sampling rate, as well as a downsampled rate of 20 Hz (achieved by block averaging) for closer comparison with DCS and iDWS. Upon visual inspection, SCOS-measured waveforms exhibited superior signal-to-noise.

For rigorous comparison, the PI of each heartbeat waveform acquired with the three modalities during baseline was computed for all subjects (see Sec. 2.3.2). As during the baseline period all runs had $\kappa_{f}^{2}>0$, we opted to not exclude the run with negative $\kappa_{f}^{2}$ during the hyperemic peak for this analysis. The average PI across the baseline periods, shown in Fig. 8(e), significantly differed across measurement techniques (p<0.001). From pairwise comparisons, the iDWS PI measurements and the downsampled (i.e., from 120 to 20 Hz) SCOS PI measurements were not significantly different (p=0.5). However, the DCS PI measurements were larger than both iDWS (p=0.01) and the downsampled SCOS PI measurements (p=0.002). Interestingly, the DCS and iDWS PI measurements were only marginally different from the unaltered (i.e., at 120 Hz) SCOS PI measurements (p=0.08 for DCS, and p=0.06 for iDWS), whereas the SCOS PI measurements at 20 and 120 Hz were significantly different from each other (p=0.002).

Finally, we computed pairwise correlations of the PI values among techniques; this analysis used the SCOS measurements downsampled (by block-averaging every six frames) to 20 Hz. These correlations are considerably weaker than those for $rBF_{i,\max}$, i.e., there was a moderate correlation between DCS and SCOS PI measurements ($r_{\mathrm{DCS}-\mathrm{SCOS}}=0.86$, p=0.001), and there were positive, but not significant, correlations between DCS and iDWS ($r_{\mathrm{DCS}-\mathrm{iDWS}}=0.61$, p=0.06) and between iDWS and SCOS ($r_{\mathrm{iDWS}-\mathrm{SCOS}}=0.36$, p=0.3). The weaker correlations among the PI measurements may be a consequence of the small sample size, probe positioning, and the greater susceptibility of PI to measurement noise (see Sec. 5).

## 5 Discussion

In this work, we compared three diffuse optical methods for measuring blood flow in biological tissues (DCS, iDWS, and SCOS). Our investigations included both *in vitro* and *in vivo* experiments to provide a wide variety of operating conditions and environmental factors to explore differences among these modalities. Arguably, the most significant difference is the SNR improvement of iDWS and SCOS over DCS. However, in practice, the optimal choice of method for the user will depend on a variety of factors including geometric requirements and constraints (spatial and temporal) associated with the investigated tissue, cost, and convenience. Thus, our primary aims were to provide strong evidence for the equivalency of flow measurements with iDWS, DCS, and SCOS and to provide information that can help researchers select an optimal blood flow monitoring modality for their specific application.

Overall, our results provide evidence that the recovered $D_{b}$ values from DCS and iDWS are in good agreement (see Figs. 4–6). The relatively small offsets of each modality from $D_{b,\mathrm{Theo}}$ in Figs. 4 and 5 can likely be explained by factors such as the limited accuracy of the FD-DOS measurement, which is expected to lead to an ∼10% error in the recovered optical properties.^74^ With FD-DOS, the recovered absorption and reduced scattering coefficients were, respectively, $\mu_{a}=0.104\pm 0.002\;{\mathrm{cm}}^{-1}$ and $\mu_{s}^{\prime }=6.30\pm 0.09\;{\mathrm{cm}}^{-1}$. Errors in the manufacturer-measured sphere diameters (i.e., 195 nm, with a ∼5% coefficient of variation) could also impact the absolute comparison with $D_{b,\mathrm{Theo}}$. The *in vitro* phantom experiment (Figs. 4 and 5) also showed the increased precision offered by iDWS and SCOS. This increase in precision can improve biomarker measurement in the clinic by revealing trends in cerebral blood flow that were previously undetectable due to noise. Moreover, the enhanced precision (and thus SNR) of iDWS and SCOS can improve depth sensitivity (via enabling measurements at larger source-detector separations) and facilitate monitoring in highly absorbing and/or highly scattering media.

During our *in vivo* arm cuff experiments, we observed a few potentially important differences that may arise when performing human or animal measurements. For instance, for one of the 10 arm cuff experiment runs, we found negative speckle contrast values during the hyperemia period post-occlusion. Negative speckle contrasts are likely related to insufficient SNR and/or a breakdown of the SCOS noise corrections. For example, although we carefully characterized our CMOS camera gain (as proposed by Ref. 66), small changes in gain can lead to large changes in the recovered hyperemic peak (as shown in Fig. 10 in the Appendix).

Small errors in the SCOS noise corrections could also potentially explain the larger $rBF_{i,\max}$ recovered in 3 out of 9 runs with SCOS [Fig. 7(b)]. These 3 runs were the lowest SNR experiments among the arm cuff experiments, and two of these runs had an order of magnitude lower $\kappa_{f}^{2}$ when compared with other experiments (i.e., $\kappa_{f}^{2}∼10^{-4}$ versus $\kappa_{f}^{2}∼10^{-3}$; refer to Table 1 from the Appendix). Further studies should investigate the errors in the SCOS noise corrections that are particularly relevant for situations with small $\kappa_{f}^{2}$ (e.g., due to high flow, large source-detector separation, high scattering, or long exposure times), where accurate noise corrections are particularly important. Importantly, all arm cuff occlusion runs (except one, which had negative speckle contrast values) recovered a speckle contrast that was at least an order of magnitude larger than the absolute value of $\kappa_{f}^{2}$ when using a noncoherent light emitting diode source in a liquid phantom (i.e., $-7\times 10^{-6}$).^52^

Another difference among the three modalities arose when determining the blood flow pulsatility index (PI) during each cardiac cycle [Fig. 8(e)]. We found statistically significant differences among the PI recovered from DCS compared with those recovered with iDWS and SCOS; iDWS and SCOS were not statistically different. The root cause of this difference could be the lower SNR of DCS, tissue heterogeneity, differences in depth sensitivity profile for each technique, or possible crosstalk introduced by fitting the iDWS $G_{2,i\mathrm{DWS}}(\tau )$ for both amplitude (A) and $BF_{i}$. Importantly, when fitting DCS for both $\beta_{\mathrm{DCS}}$ and $BF_{i}$ (as opposed to our approach of fitting for a single $\beta_{\mathrm{DCS}}$ at baseline), we observed no differences in the DCS PI measurements (p=0.3). We note further that even if DCS does not agree with iDWS and SCOS, it is not necessarily more or less accurate for recovering the PI.

In Figs. 8(a)–8(d), we also observed a few qualitative waveform shape differences across each technique. For example, the depth of the dicrotic notch is more pronounced in the average iDWS and SCOS waveforms compared with the DCS waveforms. When comparing the SCOS waveforms sampled at 20 and 120 Hz, it is also evident that the increased sampling speed afforded by the highly parallel nature of the SCOS measurements leads to differences in the waveform shape. For example, there was a clear difference in the rising edge of the pulsatile waveform (just before the systolic peak); the lower temporal resolution of the 20 Hz waveform data gives the appearance of a slower increase in the rising edge. Downsampling the SCOS data to 20 Hz also led to significantly reduced PI values when comparing SCOS at 120 Hz. These differences in PI and in the pulsatile waveform shapes are particularly important for cases where features of each waveform are considered (e.g., for estimating intracranial pressure and critical closing pressure).^44^^,^^75^^–^^78^ (Note, both iDWS and DCS can be acquired at sampling rates faster than 20 Hz, although with a potential SNR tradeoff.). These differences noted from the *in vivo* experiments highlight the importance of investigating more complex media to more closely mimic clinical measurements, i.e., instead of simple semi-infinite homogeneous optical phantoms.

In addition to the data and differences presented here, important practical features should be considered for each future experiment. With regard to system complexity, SCOS is arguably the simplest system, requiring at a minimum, a source, a camera, two optical fibers, and a computer. DCS presents some additional complexity because it requires the use of single-photon counters and an autocorrelator (which may be implemented in hardware or software). Arguably, iDWS presents the greatest level of hardware complexity due to its interferometer-based design. Alignment of the reference and sample arms must be performed carefully, and any movement in these components due to environmental factors can result in subpar optical alignment. Fiber approaches can mitigate this problem to some degree.^53^ In addition, as iDWS measures an alternating current signal, it provides the unique benefit of being unaffected by the addition of constant intensity ambient light, which could be useful in scenarios where unwanted light exposure is expected. All of these factors require careful planning in clinical scenarios, which are not as controlled as in laboratory settings.

Another important practical consideration is the complexity of the data management and data processing pipelines. DCS datasets are relatively small as DCS uses relatively few detectors and a limited number of delay times, and it does not require any calibration or noise correction steps. Combined, these factors make DCS the simplest technique for data processing and management, and perhaps the best current candidate for real-time monitoring of blood flow ($BF_{i}$). In the form employed for this study, SCOS could not achieve a real-time display of blood flow. Similarly, iDWS real-time BFI display was only possible from a few autocorrelation lags; rather, the raw data were streamed to memory without display and optimally post-processed with 4000 lags to yield BFI. These limitations are largely due to the size of the acquired datasets: for example, SCOS acquired megapixel images at a 120 Hz sampling rate and iDWS acquired data from 512 pixels every 3  μs, leading to tens (or even hundreds) of gigabytes of data generated in a few minutes. The large datasets generated by SCOS and iDWS are a challenge for long-term monitoring studies (requiring several hours or more), which at the present time is relatively easy for DCS systems.

Nevertheless, SCOS and iDWS are in their early days of development. Future more efficient processing algorithms could enable real-time processing of iDWS and SCOS data. Continued SCOS and iDWS development is also likely to lead to further improvements, e.g., the use of pulsed sources for SCOS and 1064 nm source light for iDWS have shown promise.^52^^,^^54^^,^^79^ Further enhancement of SNR from DCS has also shown promise (e.g., use of 1064 nm systems or the use of SPAD cameras^80^^,^^81^), but traditional DCS using individual point detectors is ultimately limited by its low level of parallelization.^78^^,^^80^

**Fig. 9:**
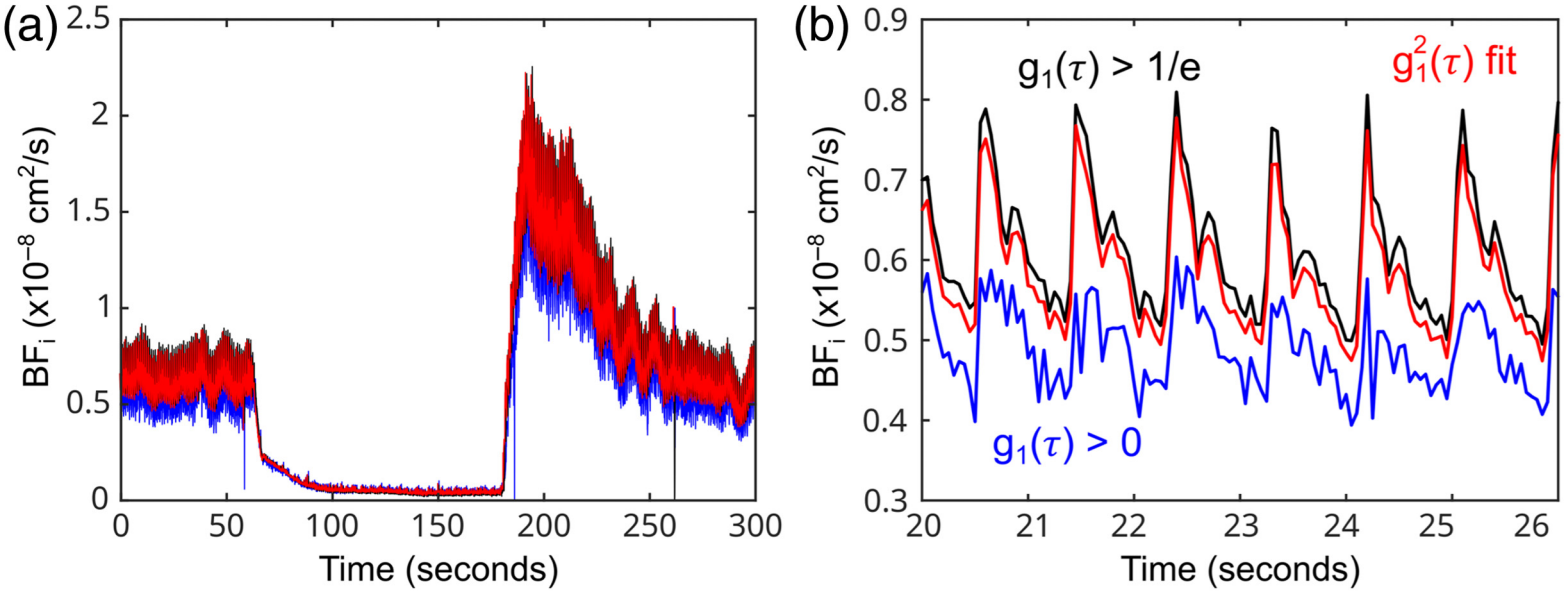
Blood flow indices (${\mathrm{BF}}_{i}$) recovered with iDWS when using different fitting methods; panel (a) shows the entire experiment, and panel (b) shows a zoomed-in look at the baseline pulsatility. Here, we show the fits using $g_{1}(\tau )$ with different cutoffs; $g_{1}(\tau )>0$ is shown in blue, and $g_{1}(\tau )>1/e$ is shown in black. The red curves exhibit results using $g_{1}^{2}(\tau )$ and a $g_{1}(\tau )>0$ cutoff (the fitting method reported in this work).

Note, a careful examination of the SNR benefits of iDWS and SCOS was not the goal of this work and would require further optimization of these techniques. It has been shown in prior work,^55^ for example, that SCOS SNR can be improved by operating at a speckle-to-pixel ratio <1. Here, we have used a speckle-to-pixel ratio of 2.3 to maximize the achievable speckle contrast.^71^ Furthermore, here we used the standard homogeneous approximation in both *in vivo* and *in vitro* measurements. Prior literature has also provided evidence that fitting measured autocorrelation functions with iDWS either $g_{1}(\tau )$ or $g_{1}^{2}(\tau )$ results in a variation in depth sensitivity. In addition to a potential depth sensitivity difference, here we also empirically found that fitting the iDWS using $g_{1}^{2}(\tau )$ yielded results that were more robust to noise (see Fig. 9 in the Appendix). Future work should further investigate the advantages and disadvantages of both approaches, particularly using well-controlled multilayer phantoms.

**Fig. 10:**
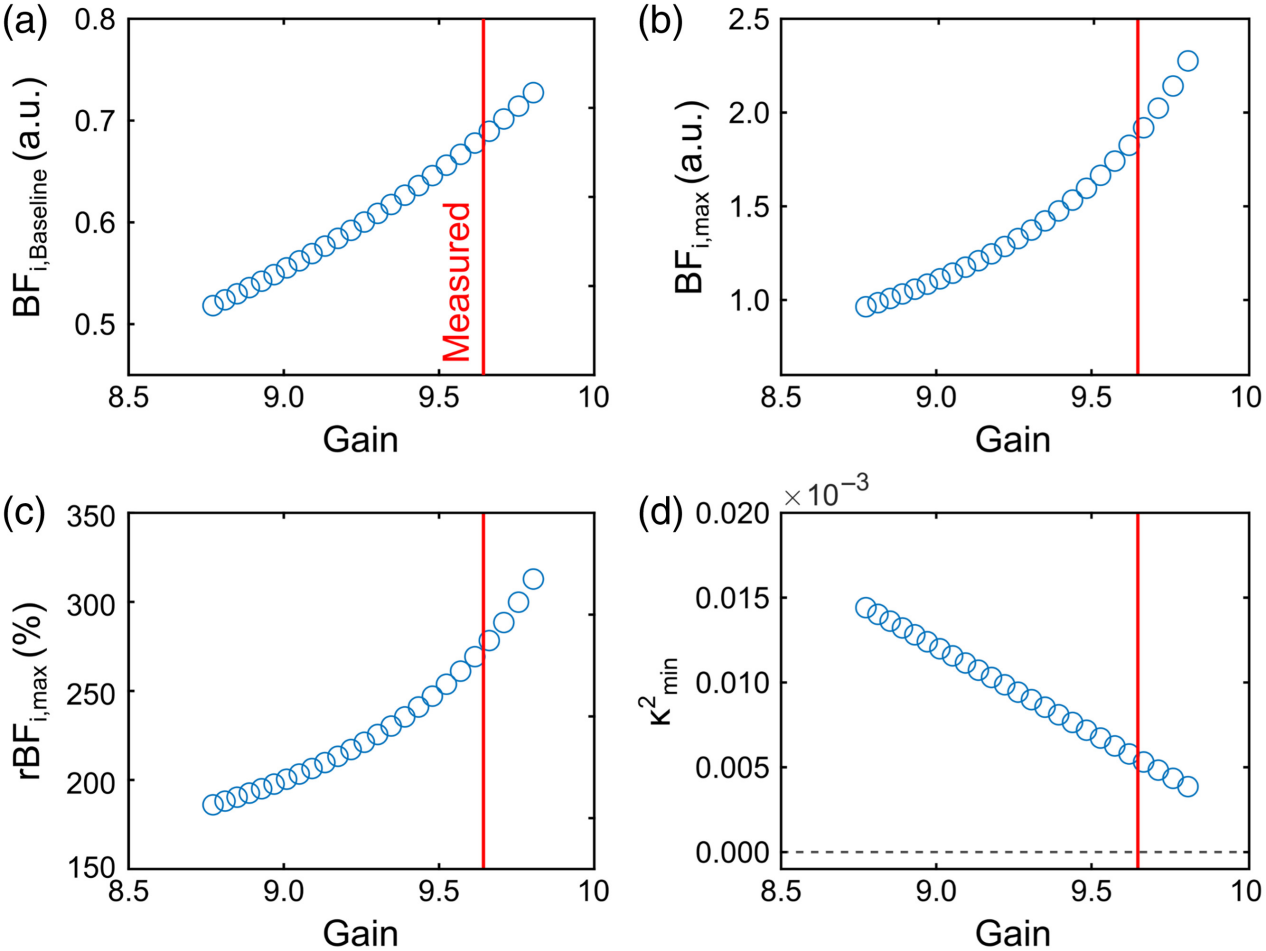
Results from a representative arm cuff experiment when artificially varying the CMOS camera gain from SCOS. Values are shown for (a) blood flow index at baseline (${\mathrm{BF}}_{i,\text{Baseline}}$), (b) blood flow index at hyperemic peak (${\mathrm{BF}}_{i,\max}$, (c) relative change in blood flow at peak flow (${\mathrm{rBF}}_{i,\max}={\mathrm{BF}}_{i,\max}/{\mathrm{BF}}_{i,\text{Baseline}}$), and (d) minimum $\kappa_{f}^{2}$ (at hyperemic peak). The red line represents the camera gain utilized in this study.

An important limitation of the present study is the use of a single exposure time for SCOS and iDWS. SNR can be further improved by increasing exposure time to increase photon collection, at the cost of a reduction in the achievable contrast for iDWS and SCOS. It should also be noted that although SCOS provided the highest SNR when compared with the other modalities, a significant contributor to its large SNR is the larger fiber used for detection and the number of pixels used for a measurement (SCOS, 1500  μm and $∼3\times 10^{6}\;\text{pixels}$; iDWS, 400  μm and 512 vertically binned pixels; and DCS, 4.4  μm and 4 SPAD “pixels”). Future SCOS studies should investigate the effects of the SCOS noise corrections for situations with smaller $\kappa_{f}^{2}$; as a reminder, small variations in the measured camera gain can drastically impact the recovered $rBF_{i,\max}$ (see Fig. 10 in the Appendix).

Regarding our *in vivo* experiments, although significant measures were taken to reduce the distance among optical detector fibers, the co-location was imperfect (center-to-center fiber distances varied between 5 and 10 mm). This will result in each modality sampling a slightly different region within the tissue. Finally, our comparisons with SCOS measurements are limited because we only employed *relative* flow measurements; future studies should develop methods that allow absolute quantification of flow with SCOS. In the future, it will be important to explore other factors that could affect the choice of technique for each clinical application, including the effects of motion artifacts, overall system stability, and depth sensitivity.

## 6 Appendix

Figure 9 provides exemplary data from a single healthy subject showing the effects of various fitting methods for iDWS. It can be seen that restricting the values of $g_{1}(\tau )$ to only those greater than 1/e improves the quality of fitted blood flow values compared to using all available decay times. Comparable results were also obtained by fitting to ${g_{1}}^{2}(\tau )$, using all available decay times, which was the chosen method for all results presented in the manuscript.

Figure 10 uses a representative arm cuff measurement from a single subject to illustrate how mis-estimation of camera gain influences blood flow recovery. An overestimation of camera gain results in an overestimation of baseline and hyperemic blood flow due to underestimation of the fundamental speckle contrast. Underestimation of camera gain causes the reverse effect, i.e., overestimation of the fundamental speckle contrast and thereby underestimation of the baseline and hyperemic blood flow values.

Table 1 provides a summary of the maximum recovered blood flow values as a percent increase from baseline, as well as the minimum recovered fundamental speckle contrast squared for all subjects and all runs of the *in vivo* forearm experiments.

**Table 1:** Maximum flow changes (${\mathrm{rBF}}_{i,\max}$) measured with DCS, iDWS, and SCOS, as well as the minimum speckle contrasts measured with SCOS across all 10 arm cuff experiments.

	*DCS*${rBF}_{i,\max}$ (%)	*iDWS*${rBF}_{i,\max}$ (%)	*SCOS*${rBF}_{i,\max}$ (%)	*SCOS* $\kappa_{f,\min}^{2}$
***Subject 1, Run 1***	*225.6*	*202.2*	*197.4*	*0.0041*
***Subject 1, Run 2***	*206.7*	*194.1*	*210.3*	*0.0018*
***Subject 2, Run 1***	*249.6*	*278.2*	*229.9*	*0.0068*
***Subject 2, Run 2***	*316.7*	*356.0*	*276.6*	*0.0062*
***Subject 3, Run 1***	*273.2*	*331.8*	*n/a*	*-0.0017*
***Subject 3, Run 2***	*221.0*	*247.2*	*604.2*	*0.0006*
***Subject 4, Run 1***	*489.2*	*608.0*	*928.0*	*0.0007*
***Subject 4, Run 2***	*520.5*	*620.5*	*863.6*	*0.0030*
***Subject 5, Run 1***	*291.6*	*293.0*	*290.0*	*0.0060*
***Subject 5, Run 2***	*266.1*	*296.1*	*268.9*	*0.0074*

## Data Availability

The data and code presented in this study are available on request from the corresponding author.
